# Proteomic and Secretomic Response of an African *Armillaria* Species to Iron

**DOI:** 10.1021/acs.jproteome.5c00979

**Published:** 2026-02-20

**Authors:** Deborah L. Narh, Brenda D. Wingfield, Martin P. A. Coetzee

**Affiliations:** † Department of Biochemistry, Genetics and Microbiology, Forestry and Agricultural Biotechnology Institute (FABI), Faculty of Natural and Agricultural Sciences, 728875 University of Pretoria, Pretoria 0002, South Africa

**Keywords:** basidiomycete, fungal proteomics, iron homeostasis, phytopathogen, proteogenomics, secondary metabolite gene clusters, secretomics

## Abstract

*Armillaria* species have attracted considerable research interest, because they are widely distributed, mostly plant-pathogenic, and exhibit unique characteristics. Abiotic factors influence intra- and interspecies variations in pathogenicity and/or virulence of these fungi. However, the mechanisms involved in causing these variations are not well understood. Iron is an indispensable element in several molecular and biological processes. Yet, excessive abundance of iron can be toxic to organisms due to Fenton-like reactions. This study aimed to gain insights into the type and extent of iron-responsive proteomic and secretomic changes in *Armillaria* sp. strain CMW4456 cultured in liquid media supplemented with iron using a multiomics approach. Significant iron-dependent alterations of proteins involved in metabolism and growth were observed in the proteomes and secretomes. Iron supplementation at 100 μM did not elicit an oxidative stress response by the fungus. Our analyses revealed three putative siderophore biosynthetic gene clusters (BGCs) in the genome and expression of proteins encoded by some BGC genes in the proteome. This knowledge contributes to a better understanding of the mechanisms employed by an *Armillaria* sp. in response to iron, gives insights into possible modes for inhibiting or attenuating the pathogenicity and/or virulence of *Armillaria* spp., and can be valorized for more biotechnological applications.

## Graphical abstract

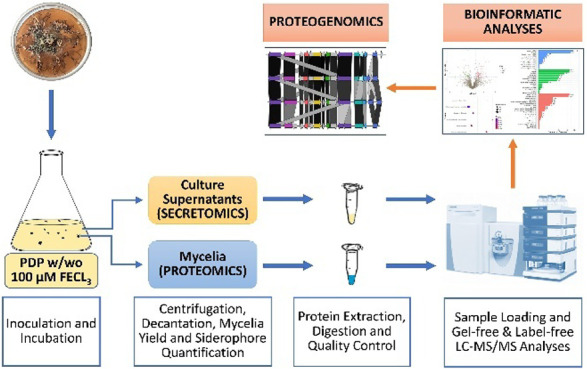

## 1 Introduction

*Armillaria* species have a worldwide geographic distribution and inhabit a wide range of ecological niches.^1,2^ Most *Armillaria* spp. are classified as facultative necrotrophs because they have both saprophytic and pathogenic phases.^2^ These species threaten the health of woody plants in native and managed timber stands and agronomic plantations in areas where they are established.^1,3^

Various factors influence the lifestyle of the *Armillaria* species. Among these, growth mechanisms, as well as the expression of genes responsible for the production of plant cell wall degrading enzymes (PCWDEs), secondary metabolites, and other pathogenicity-related gene products, are factors responsible for their lifestyle.^4–6^ The switch from saprophytic to pathogenic lifestyle and vice versa, and host invasion by these fungi are influenced by factors such as intraspecies variation, forest management systems, age and state of the hosts (e.g., diseased, stressed, or healthy), elevation, as well as the presence of other organisms in the rhizosphere of the host.^7–9^ The mechanisms underlying these effects are not clearly understood.

Abiotic factors such as metal concentration and/or availability,^10–15^ drought or water activity,^16^ carbon dioxide concentration,^17^ salinity,^14,18^ and heat^19,20^ elicit various proteomic responses in organisms. Among metals, iron homeostasis is vital because iron is an indispensable element in several molecular and biological processes in many organisms.^21^ Additionally, animal–microbe, microbe–microbe, and plant–microbe interactions have been shown to be influenced by iron uptake and transport mechanisms exhibited by the respective organisms.^22–25^ Yet, an abundance of internal iron is toxic to organisms due to the occurrence of Fenton-like reactions that can produce both hydroxyl radicals and higher oxidation states, which may cause biological damage.^26,27^ Consequently, investigations into the iron-dependent proteomes and/or secretomes of organisms including the opportunistic fungal human pathogen, *Aspergillus fumigatus*,^12,28^ and other microorganisms^13,29,30^ have been conducted. These studies have highlighted various, often extensive, changes in the proteomes or secretomes of the studied organisms. The reported changes in these proteomes and secretomes have often been associated with mechanisms utilized for iron homeostasis. These mechanisms include the assembly and/or synthesis of antioxidant enzymes, metal-chelating proteins or molecules, and free radical scavengers.^28^ The reader is referred to the review article by Misslinger et al.^28^ for details about some of these mechanisms.

Unusual iron-dependent growth and siderophore (low-affinity iron-chelating protein molecules) biosynthesis in strains of various species of *Armillaria* has previously been reported.^31^ This suggested that *Armillaria* spp. have species-independent atypical iron requirements.^31^ Three secondary/specialized metabolite gene clusters (SMGCs) putatively responsible for siderophore biosynthesis have also been reported in the genomes of *Armillaria* and other species in the Physalacriaceae.^31,32^ In the present study, we applied *in vitro* bioassays, the gel-free and label-free LC-MS/MS bottom-up shotgun proteomics approach, and proteogenomics. The study aimed to gain insight into the type and extent of iron-responsive proteomic and secretomic changes exhibited by cultures grown in liquid medium of one isolate of an *Armillaria* sp. from Zimbabwe for which the genome was sequenced earlier,^33^ and to investigate the presence, conservation, and/or expression of genes of the previously reported putative siderophore biosynthesis SMGCs^31,32^ in the genome. The knowledge generated will broaden our understanding of iron-dependent mechanisms employed by *Armillaria* spp. and can be used in the future to develop better control strategies against pathogenic members of this genus and for other applications.

## 2 Experimental Section

All reagents used in this study were of analytical grade or equivalent and were purchased from commercial suppliers.

### 2.1 Strain Used, Culture Conditions, and Siderophore Biosynthesis Assay

The studied isolate, *Armillaria* sp. strain CMW4456, belongs to African Group B (*sensu* Coetzee et al. 2005) and was obtained from *Brachystegia utiliz* at Stapleford, Zimbabwe.^34,35^ The genome of this isolate has been sequenced by our team.^33^

Plastic Petri dishes were used, and glassware was washed with HCl (6 M) followed by a 3 time rinse with ddH_2_0 to avoid iron contamination.^36,37^ Cultures were grown on potato dextrose peptone agarose (PDPA; 24 g/L potato dextrose, 2 g/L peptone, and 10 g/L agarose) in 6.5 cm disposable Petri dishes. Agarose was used instead of agar to reduce iron contamination in the medium.^38^ For iron-deplete conditions in liquid medium, agarose was omitted from PDPA to obtain PDP–. Iron-replete conditions were attained by supplementing PDP– with 100 μM FeC1_3_·6H_2_O (PDP+). The liquid media (100 mL) were inoculated with 1 cm^2^ actively growing culture. There were three biological replicates per treatment. All cultures were incubated at 25 ± 2 °C in the dark for 3 weeks. Cultures in liquid medium were swirled to mix every week. At the end of the incubation period, mycelia were separately harvested from these cultures by centrifugation (10,000 × *g* for 20 min at 4 °C), followed by decantation of the culture supernatant into fresh Eppendorf tubes on ice. Harvested mycelia were washed three times with sterile distilled water, and the fresh weights of the cultures were determined. The mycelia and supernatants were used for the proteomic and secretomic studies.

Siderophore biosynthesis was also determined in liquid cultures using the modified chrome azurol S (CAS) assay solution with the microtiter method described by Alexander and Zuberer.^39^ The percentage siderophore units (psu) were calculated as follows using the formula reported by Payne:^40^

$$\mathrm{Siderophore production}\;(\mathrm{psu})=\frac{\left(A_{r}-A_{s}\right)\times 100}{A_{r}}$$

where *A*_r_ = absorbance of reference [CAS solution and uninoculated broth (control)], and *A*_s_ = absorbance of sample (CAS solution and cell-free supernatant of sample). Statistical significance of siderophore production by the samples cultured under the two study conditions was calculated using t-Test: Paired Two Samples for Means in Microsoft Excel.

### 2.2 Protein Extraction

Protein extraction from mycelia was performed according to previously described methods,^12,41^ but with some modifications. In brief, mycelia obtained from both treatments were suspended in 6 mL of cold lysis buffer (25 mM Tris-HCl, 6 M GdnHCl, 10 mM DTT, pH 8.6) in a tube containing a ceramic bead and homogenized. The samples were then transferred to new tubes and sonicated (cycle 6, 10 s, power 10%) on ice. All lysates were clarified twice by centrifugation (10,000 × *g* for 20 min at 4 °C) and transferred to clean tubes on ice. The samples were then brought to 15% (w/v) trichloroacetic acid (TCA), swirled on a rotor on ice for 30 min to precipitate proteins. Proteins were washed three times with ice-cold acetone with centrifugation (10,000 × *g* for 10 min at 4 °C). Pellets were resuspended in UT buffer (6 M urea, 2 M thiourea, and 0.1 M Tris-HCl, pH 8).

### 2.3 On-Bead Digestion and LC-MS/MS Analyses

Sample digestion, peptide concentration determination, and LC-MS/MS analyses were conducted at the Proteomics Spectrometry Unit, Central Analytical Facilities of Stellenbosch University (Stellenbosch, South Africa).

#### 2.3.1 Sample Preparation for LC-MS/MS

Buffer change and sample cleanup were performed preceding on-bead digestion by resuspending all sample solutions in Tris buffer containing 5 mM tris­(2-carboxyethyl)­phosphine (TCEP; Fluka). The samples were then sonicated for 30 s in a cooled sonic bath, followed by vortexing at a high speed for 30 s. This process was repeated five times. For on-bead trypsin digestion of protein extracts, samples were resuspended in 100 mM Tris-HCl (Sigma) containing 100 mM NaCl and 1% SDS (Sigma) before reduction with 5 mM TCEP in 100 mM Tris buffer for 1 h at 60 °C. Cysteine residues were thiomethylated with 20 mM S-methylmethanethiosulfonate (Sigma) in 100 mM triethylammonium bicarbonate (TEAB; Thermo Scientific) for 30 min at room temperature. Samples were then diluted 2-fold with binding buffer (100 mM ammonium acetate, 30% acetonitrile, pH 4.5). The protein solution was added to MagResyn HILIC magnetic particles (Resyn Biosciences) prepared according to the manufacturer’s instructions and incubated overnight at 4 °C to bind. After binding, the supernatant was removed, and the magnetic particles were washed twice with washing buffer (95% acetonitrile (ACN; Romil)). The magnetic particles were then suspended in 25 mM ammonium bicarbonate containing trypsin (New England Biosystems) in a final ratio of 1:50. After an 18 h incubation at 37 °C, the peptides were extracted once with 50 μL of 15% trifluoroacetic acid (TFA; Sigma). The samples were then dried and resuspended in 30 μL ddH_2_0 for peptide concentration determination. The peptide concentration of each biological replicate per sample was determined using Pierce Quantitative Peptide Assays & Standards (Thermo Scientific) according to manufacturer’s instructions.

#### 2.3.2 LC-MS/MS Analysis

Liquid chromatography (LC) was performed on a Dionex Ultimate 3000 RSLC nano LC (Thermo Scientific; Massachusetts, USA) equipped with a C_18_ trapping column (Thermo Scientific; 5 mm × 300 μm, 5 μm; pore size 100 Å) and a CSH 25 cm × 75 μm, 1.7 μm particle size C_18_ analytical column (Waters) according to the protocol described by Ellero et al.^42^ with some modifications. The samples were loaded onto the trap column using loading solvent (2% acetonitrile:water; 0.1% formic acid (FA)) at a flow rate of 2 μL/min for 5 min from a temperature-controlled autosampler, set at 7 °C, before the sample was eluted onto the analytical column. The solvent system employed for elution was: Solvent A, 2% acetonitrile:water; 0.1% FA, and Solvent B, 100% acetonitrile:water. The flow rate for elution was set to 300 nL/min, and the gradient was generated as follows: 5–30% Solvent B over 60 min and 30–50% Solvent B from 60–80 min. Chromatography was performed at 45 °C, and the outflow was delivered to the mass spectrometer.

Tandem mass spectrometry (MS/MS) was performed using a Thermo Scientific Fusion Orbitrap Mass Spectrometer equipped with a Nanospray Flex ionization source. The sample was introduced through a stainless-steel nanobore emitter. Data were collected in positive mode with the following parameters: spray voltage = 1.8 kV, ion transfer capillary = 275 °C. Polysiloxane ions at *m*/*z* = 445.12003 were used to calibrate the spectra internally. MS1 scans were performed in profile mode using the Orbitrap detector set at 120,000 resolution over the scan range 375–1500, with the Automatic Gain Control (AGC) target set at 4 × 10^5^ and a maximum injection time of 50 ms. MS2 acquisitions in centroid mode were performed using monoisotopic precursor selection for ions with charges +2 to +7 and an error tolerance set to ±10 ppm. Precursor ions were excluded from fragmentation once for 60 s. Precursor ions were selected for fragmentation in higher-energy collisional dissociation (HCD) mode using the quadrupole mass analyzer with HCD energy set to 30%. Fragment ions were detected in the Orbitrap mass analyzer set to 30,000 resolution. The AGC target was set to 5 × 10^4^ and the maximum injection time to 100 ms.

### 2.4 Spectra Processing, Bioinformatic, and Proteogenomic Analyses

#### 2.4.1 Spectra Processing and Bioinformatic Analyses

Data retrieved from LC-MS/MS analyses of biological replicates from mycelia obtained from PDP– and PDP+ represented the proteome under iron-deplete (Mde) and iron-replete (Mre) conditions, respectively. Supernatants obtained from PDP– and PDP+ represented the secretome under iron-deplete (Sde) and iron-replete (Sre) conditions, respectively. Data processing and analyses were conducted at BGI Genomics (https://www.bgi.com/global) and are outlined below.

The thermo.raw files generated by the mass spectrometer were imported into MaxQuant 1.6.2.3 ^43^ and processed using the integrated search engine of MaxQuant, Andromeda.^44^ MaxQuant was also used to extract peak areas and calculate protein quantitation values. Quantitative analysis was performed based on the peak intensity, peak area, and LC retention time of the peptides related to the first-order mass spectrometry (MS1). A series of statistical analyses and quality control steps were also performed to obtain significant identification results using the following parameters: Enzyme = Trypsin, Peptide_Mass_Tolerance = 10 ppm, Fragment_Mass_Tolerance = 0.02 Da, Minimal peptide length = 7, PSM-level FDR = 0.01, Protein-level FDR = 0.01, Fixed modification = Carbamidomethyl (C), Variable modifications = Oxidation (M), Acetyl (Protein N-term), Deamidated (NQ), and Gln → pyro-Glu, and Database = 1253_all_filtered.fasta (83,019 sequences). The data were searched using the UniProt Protein Database, Augustus annotation of the genome of strain CMW4456 (accession number JANDKJ000000000.1),^33^ and protein sequences of *A. mellea* strain DSM 3731 (sample code Armme1_1)^5^ and *A. ostoyae* strain C18/9 (sample code Armosto1)^4^ obtained from JGI.^45^ Protein quantification and difference analysis were conducted on the set comparison groups (i.e., Mre vs Mde and Sre vs Sde). The multiples of differences in the proteins in each comparison group were calculated. A significance test was performed using Welch’s *t* test. Protein abundances with a fold change (FC) >1.5 and *p*-value <0.05 were considered as differentially expressed proteins (DEPs). Unique and shared protein expression profiles between the Mre vs Mde and Sre vs Sde comparison groups were evaluated using Venny version 2.1.^46^

Functional annotations such as Gene Ontology (GO),^47^ euKaryotic Ortholog Groups (KOG),^48^ and Kyoto Encyclopedia of Genes and Genomes (KEGG)^49^ for pathway analyses were automatically performed based on the identified DEPs. GO functional annotation analysis included two parts: protein2go and go2protein. For each protein, a list of identities (IDs) and all corresponding GO functions were given in the protein2go results. In terms of go2protein, for the GO entries involved in the three ontology classifications (i.e., biological process, cellular component, and molecular function), the identities and number of proteins of all of the corresponding proteins were listed, and the GO entries without the corresponding proteins were omitted. KOGs are databases for the orthologous classification of proteins. Each KOG entry contains a series of orthologous proteins or paralogs. The analysis compared the identified proteins with the KOG database, predicted the possible functions of these proteins, and performed functional classification statistics on the identified proteins. *In vivo*, different proteins coordinate biological behaviors, and pathway-based analysis therefore helps further understand their biological functions. KEGG is the main public database on pathways^49^ and was used to annotate molecular networks (pathways) of the DEPs.

#### 2.4.2 Proteogenomics for Detection of Siderophore Biosynthetic Gene Clusters (BGCs) and BGC Gene Expression

The draft genome sequence of CMW4456 (accession number: JANDKJ000000000)^33^ was investigated for NRPS and NIS synthetase gene clusters reported by our group for other strains of *Armillaria* ^31,32^ and to link the proteins encoded by the genes in the gene clusters to the proteins detected in the present study. This was achieved by using the previously described comparative genomics methods^31,32^ but with some modifications. Synteny analyses of the detected siderophore biosynthetic gene clusters (BGCs), compared to some of the previously reported putative siderophore BGCs recorded in other *Armillaria* genomes^31,32^ were conducted using the Clinker tool of the online CompArative GEne Cluster Analysis Toolbox (CAGECAT) with the identity threshold set at 0.3.^50^ The siderophore BGCs that the putative siderophore BGCs identified in the genome of strain CMW4456 were compared to the siderophore BGCs extracted from the genomes of *A. borealis* strain FPL87.14 v1.0 (Armbor1), *A. cepistipes* strain B5 (Armcep1), *A. fumosa* strain CBS 122221 v1.0 (Armfum1), *A. mellea* strain ELDO17 v1.0 (Armme1), and *Desarmillaria ectypa* strain FPL83.16 v1.0 ((Des)­Armect1). All these genomes and their respective RNA sequences were downloaded from JGI MycoCosm.^45^ (Des) has been included in the original genome code of Armect1 to indicate that this species belongs to the sister genus *Desarmillaria*.^51^ The (Des)­Armect1 genome was used with permission from Dr. László G. Nagy. The proteome and secretome obtained in Section 2.4.1 are manually searched for expression of the proteins encoded by the genes in the putative siderophore BGCs.

## 3 Results

### 3.1 Culture Yield and Siderophore Biosynthesis

There were differences in the mycelia growth of strain CMW4456 in the two treatments. The culture fresh weight of strain CMW4456 was almost 2 times higher when grown in liquid potato dextrose peptone medium with iron supplementation (PDP+; 0.501–0.556 g) compared to when grown in the same medium without iron supplementation (PDP–; 0.311–0.376 g). This suggests that iron supplementation of the growth medium at 100 μM enhanced the mycelia growth of the strain by almost 2-fold under the study conditions.

Additionally, we recorded siderophore biosynthesis under both iron-replete and iron-deplete conditions. The culture grown in PDP+ and PDP– yielded percentage siderophore units (psu) of 42.94 ± 1.39 and 42.77 ± 0.5, respectively. The difference in siderophore biosynthesis between the two treatments was not significant (*p* = 0.47), suggesting that supplementation of the growth medium with 100 μM FeCl_3_ was insufficient to inhibit siderophore biosynthesis by the fungus.

### 3.2 General Characteristics of the Proteome and Secretome under the Experimental Conditions

As expected, there were more identified proteins in the proteomes than in the secretomes. We identified 2,360–2,453 and 2,498–2,509 proteins in the proteomes under iron-deplete (Mde) and iron-replete (Mre) conditions, respectively, and 379–463 and 393–448 proteins in the secretomes under iron-deplete (Sde) and iron-replete (Sre) conditions, respectively. We detected 327 and 555 proteins in the medium only with (Cre) and without (Cde) iron supplementation, respectively. The molecular weights of most of the detected proteins were in the range of 30–40 kDa.

The entire data set is presented as principal component analysis (PCA) in which replicate samples are reduced into their corresponding sample groups (Figure 1). The positioning of samples in the PCA depicts the variability of the samples relative to one another. Results of the PCA indicated that the combined variance between components 1 (PC1) and 2 (PC2) amounts to 75.38%, with PC1 contributing 71.11% to the variance. The PCA plot of proteins of *Armillaria* sp. strain CMW4456 demonstrated distinct differences in the proteomes and secretomes under both conditions. The media only (Cde and Cre) also grouped separately. The results also showed good repeatability of each biological replicate in the same sample group (i.e., proteome and secretome).

**Figure 1:**
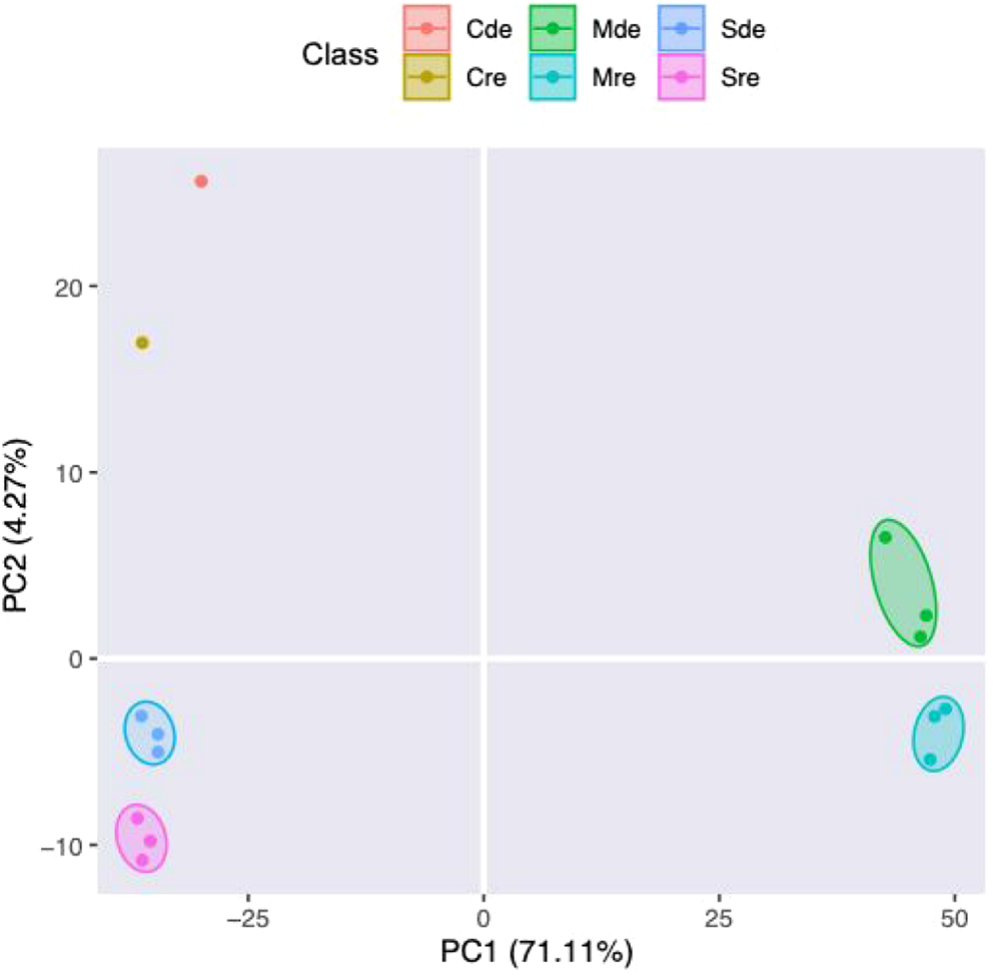
Score plot of principal component analysis (PCA) of samples comparing components 1 and 2. PC1 = first principal component; PC2 = second principal component. Cde = iron-deplete medium (i.e., uninoculated medium without the addition of FeCl_3_; PDP−); Cre = iron-replete medium (i.e., uninoculated medium with added 100 μM FeCl_3_; PDP+); Mde = iron-deplete mycelia (i.e., iron-deplete proteome); Mre = iron-replete mycelia (i.e., iron-replete proteome); Sde = iron-deplete supernatant (i.e., iron-deplete secretome); Sre = iron-replete supernatant (i.e., iron-replete secretome). Corresponding color-coded oval shapes indicate clustering of the respective samples. *n* = 3 per sample group. Controls for each treatment were uninoculated media (Cde and Cre; *n* = 1 each).

The proteomes and secretomes were altered with iron supplementation. Proteomes obtained with (Mre) and without (Mde) iron supplementation revealed 167 differentially expressed proteins (DEPs). Among these, 60 proteins were down-expressed (decreased in abundance), and 107 proteins were up-expressed (increased in abundance) in Mre compared with Mde (Figure 2A). Secretomes obtained under the two conditions revealed 15 DEPs between the iron-replete (Sre) and iron-deplete (Sde) conditions, 13 of which were down-expressed in Sre compared to Sde (Figure 2B). Proteins shared between the two comparison groups were all nonsignificantly altered proteins (265 proteins; Figure 2C). The list of up-, down-, and nondifferentially expressed proteins in both the proteome and secretome for the entire data set for the two comparison groups as well as for the unique and shared proteins to and/or between the two comparison groups can be found in Material S1.

**Figure 2:**
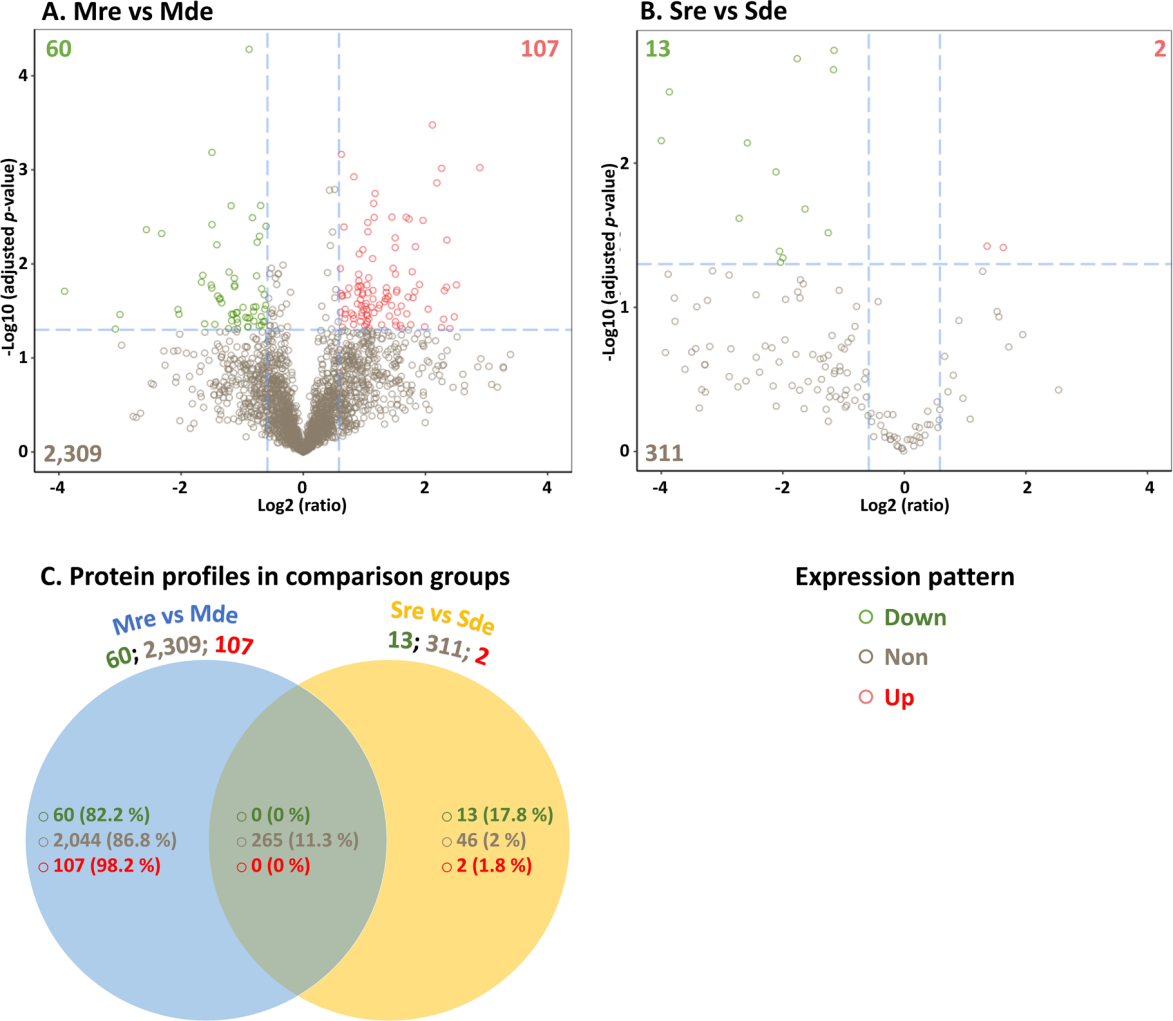
Volcano plots and Venn diagram showing changes in protein expression in the respective comparison groups. (A) Proteome under iron-replete compared to iron-deplete conditions (Mre vs Mde) and (B) secretome under iron-replete compared to iron-deplete conditions (Sre vs Sde). The *x*-axes are the protein fold change (log 2), and the *y*-axes are the corresponding −log 10 (adjusted *p*-value). (C) Unique and shared protein expression profiles across the analyzed dimensions. For each comparison group, the numbers following the comparison group are presented as the total number of recorded proteins for the respective expression patterns. The values presented in brackets represent the percentage of the data in the union of the recorded proteins in the two comparison groups. In the figures, the green circles are significantly down-expressed proteins, the red circles are significantly up-expressed proteins, and the brown circles are proteins with nonsignificantly altered expression. Corresponding color-coded numbers are the numbers of down-expressed, up-expressed, and nonsignificantly altered proteins, respectively, under iron-replete compared to iron-deplete conditions.

### 3.3 Qualitative Variations in Differentially Expressed Proteins

#### 3.3.1 Gene Ontology Classification of DEPs

Gene Ontology (GO) annotation and functional classification of the DEPs identified in the evaluated samples revealed that the proteome and secretome of *Armillaria* sp. strain CMW4456 were altered in response to iron supplementation. The DEPs were classified into biological process (BP), cellular component (CC), and molecular function (MF) for Mre versus Mde and Sre versus Sde comparison groups (Figure 3A and B, respectively).

**Figure 3:**
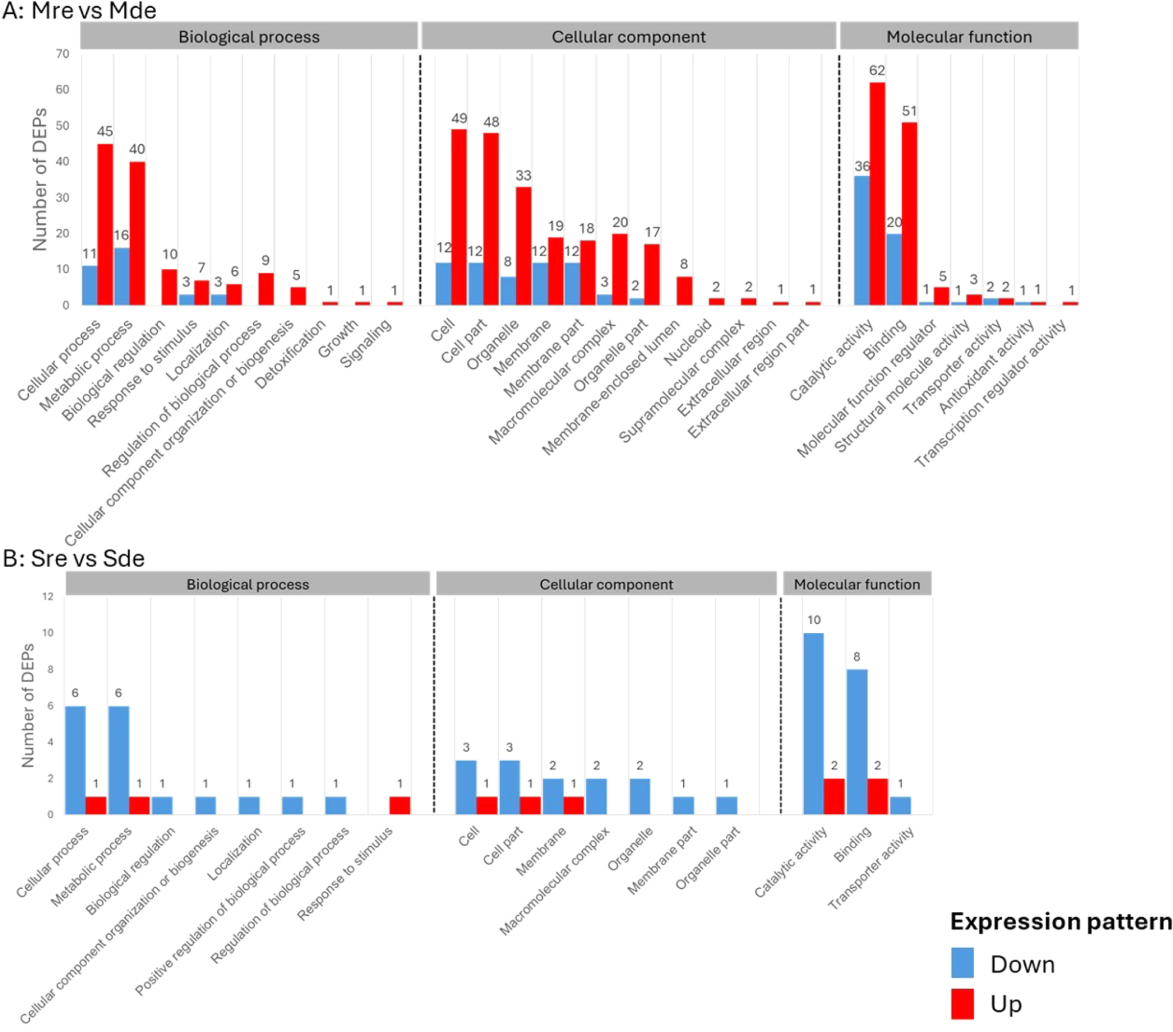
Significantly enriched Gene Ontology annotation and functional classification of the DEPs. (A) Proteome under iron-replete compared to iron-deplete conditions (Mre vs Mde) and (B) secretome under iron-replete compared to iron-deplete conditions (Sre vs Sde). The *x*-axes represent the GO annotation entries, classified into Biological Process (BP), Cellular Component (CC), and Molecular Function (MF). The *y*-axes represent the number of differentially down- or up-expressed proteins (blue and red bars, respectively). Numbers outside the bars are the number of down- or up-expressed proteins.

The GO functional enrichment analysis of DEPs in the Mre vs Mde comparison group classified 623 DEPs into 29 GO terms. There were 10 BP, 12 CC, and 7 MF GO terms. The 3 main GO annotations in the proteomes in the BP classification were “cellular process” (11 down- and 45 up-expressed DEPs), “metabolic process” (16 down- and 40 up-expressed DEPs), and “biological regulation” (0 down- and 10 up-expressed DEPs). The conspicuous terms under CCs for this comparison group were “cell” (12 down- and 49 up-expressed DEPs), “cell part” (12 down- and 48 up-expressed DEPs), and “organelle” (8 down- and 33 up-expressed DEPs), while the conspicuous GO terms classified under MFs were “catalytic activity” (36 down- and 62 up-expressed DEPs) and “binding” (20 down- and 51 up-expressed DEPs) (Figure 3A).

In the Sre vs Sde comparison group, there were fewer GO terms to which the DEPs were annotated (18 GO terms), with 8, 7, and 3 terms classified under BP, CC, and MF, respectively (Figure 3B). As found in the proteomes, GO terms in the secretomes in the BP classification included “cellular process” (6 down- and 1 up-expressed DEPs) and “metabolic process” (6 down- and 1 up-expressed DEPs). Those in the CC classification were “cell” and “cell part” (3 down- and 1 up-expressed DEPs for both functions), while the most abundant GO terms in the MF classification were “catalytic activity” (10 down- and 2 up-expressed DEPs) and “binding” (8 down- and 2 up-expressed DEPs) (Figure 3B).

#### 3.3.2 KOG Classification of Differentially Expressed Proteins

KOG functional classification of the DEPs also revealed differences in the functional annotation of the DEPs in the proteome and secretome under the experimental conditions. There were 22 functions identified among the DEPs detected in proteomes obtained under iron-replete compared with iron-deplete conditions (Mre vs Mde; Figure 4A). “General function prediction only”, belonging to the “poorly characterized” category, accounted for 32 proteins out of the 193 DEPs. There was a total of 72, 35, and 46 DEPs assigned to the main KOG classifications, “metabolism”, “information storage and processing”, and “cellular processes and signaling”, respectively. Under the “metabolism” category, 20, 14, and 13 DEPs were assigned to “energy production and conversion”, “lipid transport and metabolism”, and “amino acid transport and metabolism”, respectively. Additionally, 10 DEPs under this category were assigned to “secondary metabolite biosynthesis, transport and catabolism” and “carbohydrate transport and metabolism”. Under the “information storage and processing” category, “translation, ribosomal structure and biogenesis” (11 DEPs) and “RNA processing and modification” (9 DEPs) were the most abundant KOG functions. Most of the DEPs (23 proteins) classified under the “cellular processing and signaling” category function in “post-translational modification, protein turnover, chaperons”, followed by 9 DEPs functioning in “signal transduction mechanisms”.

**Figure 4:**
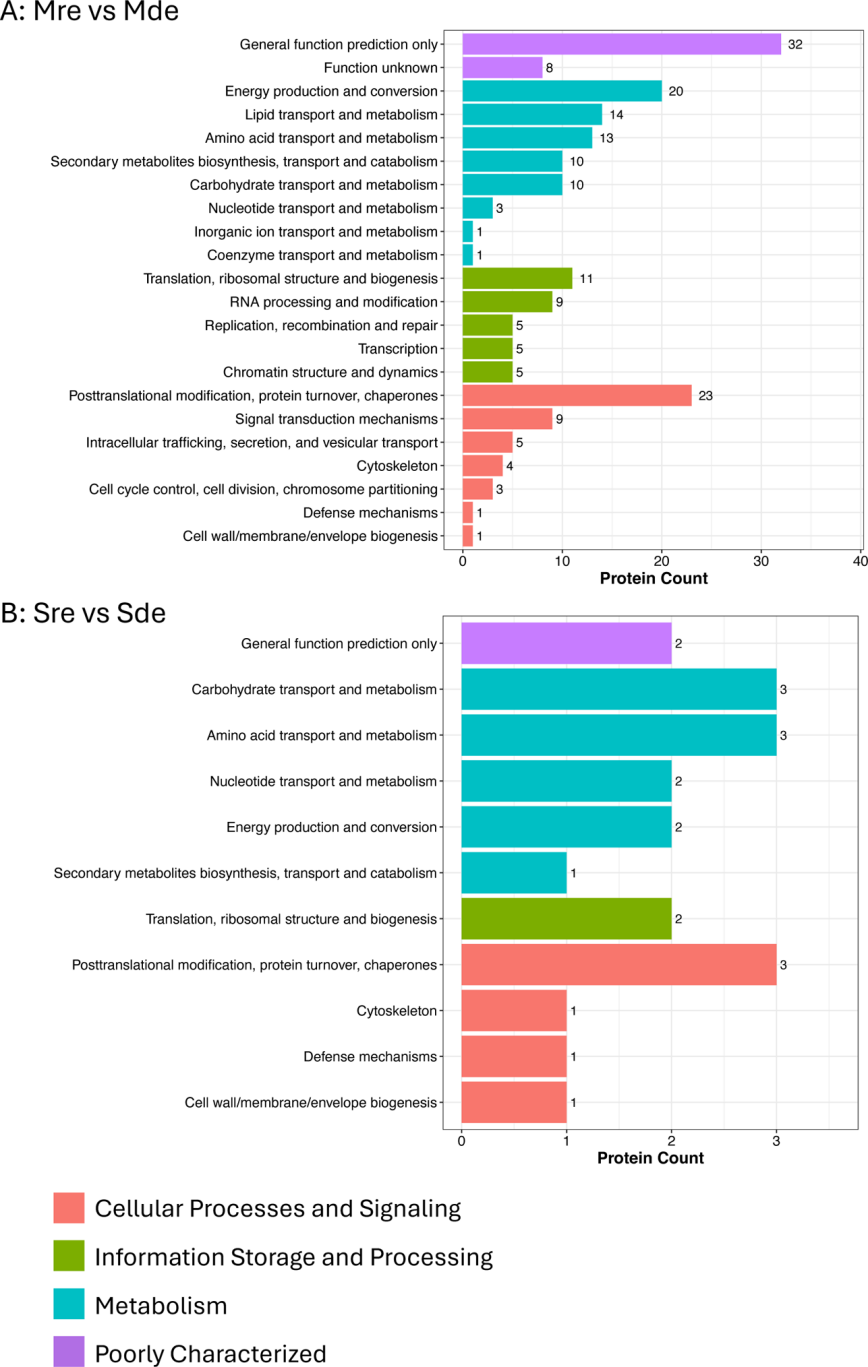
KOG classification of differentially expressed proteins. (A) Proteome under iron-replete conditions compared to iron-deplete conditions (Mre vs Mde) and (B) secretome under iron-replete conditions compared to iron-deplete conditions (Sre vs Sde). The *x*-axes display protein count, and the *y*-axes display KOG terms grouped under cellular processes and signaling, information storage and processing, metabolism, and poorly characterized terms. Numbers outside the bars are the number of DEPs.

The 21 DEPs of secretomes obtained under iron-replete compared to iron-deplete conditions (Sre vs Sde) were annotated to 11 functions under all 4 KOG classifications (Figure 4B). Two DEPs each were assigned to “general function prediction only” and “translation, ribosomal structure and biogenesis” under the “poorly characterized” and “information storage and processing” categories, respectively. Most of these DEPs (11 proteins) were assigned to the KOG term, “metabolism”, followed by 6 DEPs assigned to “cellular processing and signaling”. Under the “metabolism” category, 3 DEPs each function in “carbohydrate transport and metabolism” and “amino acid transport and metabolism”. Three DEPs under the “cellular processing and signaling” category function in “post-translational modification, protein turnover, chaperons”.

#### 3.3.3 KEGG Pathway Annotation

The DEPs were mapped to KEGG pathways to explore the biological functions of the DEPs with regard to the different sample comparison groups (Figure 5). Between Mre versus Mde (Figure 5A) and Sre versus Sde (Figure 5B) comparison groups, 8 and 4 KEGG pathways, respectively, were found to have significantly different enrichment patterns. The pathways significantly enriched in proteomes under iron-replete compared to the iron-deplete conditions (Mre vs Mde) had Rich Factors between 0.3 and 0.4. Eight of these DEPs function in “pentose phosphate pathway” (*p*-value = 0.000). All the other pathways had Rich Factors less than 0.2. These included “biosynthesis of secondary metabolites” (30 DEPs, lowest Rich Factor, *p*-value = 0.043), “carbon metabolism” (16 DEPs, second lowest Rich Factor, *p*-value = 0.006), and “citrate cycle (TCA cycle)” (6 DEPs, third lowest Rich Factor, *p*-value = 0.036).

**Figure 5:**
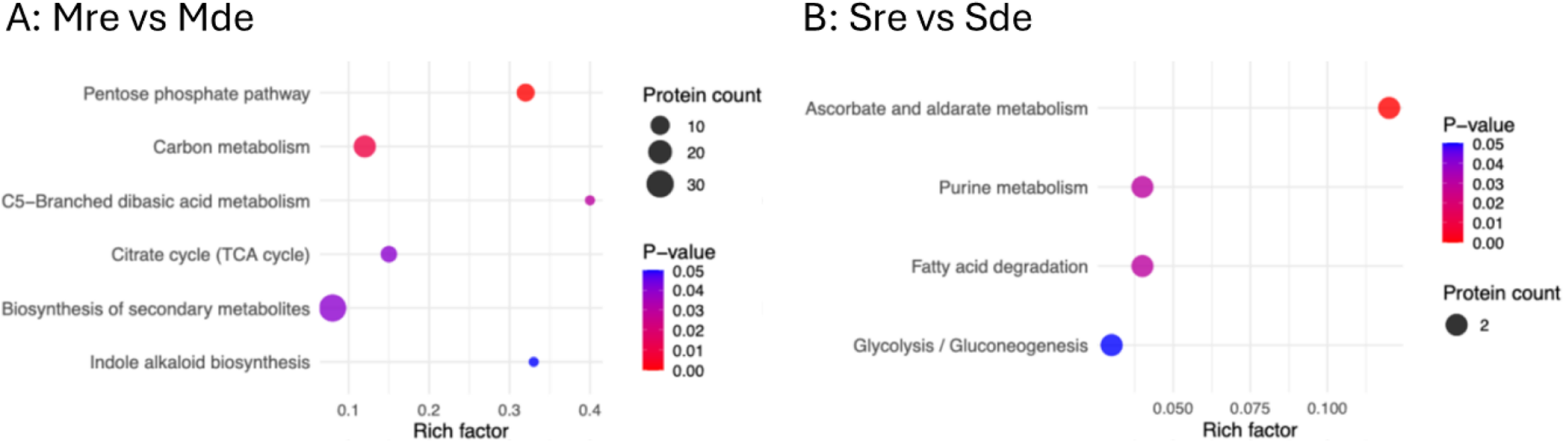
Bubble diagrams of KEGG pathway enrichment of DEPs showing the pathways in which the DEPs are significantly enriched: (A) proteome under iron-replete compared to iron-deplete conditions (Mre vs Mde) and (B) secretome under iron-replete compared to iron-deplete conditions (Sre vs Sde). The Rich Factor (also known as enrichment factor; *x*-axes) is the ratio of the number of DEPs annotated to the pathway to all the proteins identified in the pathway. The greater the Rich Factor, the higher the degree of enrichment. The *y*-axes are the pathways enriched in DEPs. The bubble sizes represent the number of DEPs annotated to the pathway, and the depth of the bubble color represents the adjusted *p-*value.

In the secretome, under iron-replete compared to the iron-deplete conditions (Sre vs Sde), there were 2 DEPs each in all the 4 reported pathways, all of which were down-expressed. The pathways with the lowest and highest Rich Factors were shown to function in “glycolysis/gluconeogenesis” (Rich Factor <0.05; *p*-value = 0.049) and “ascorbate and aldarate metabolism” (Rich Factor >0.1; *p*-value <0.004), respectively.

### 3.4 Detected Siderophore BGCs and Expression of Genes in Detected BGCs

A BGC belonging to the nonribosomal peptide synthetase (NRPS)-dependent siderophore synthetase pathway (NDSS; Figure S1a) and two BGCs belonging to the NRPS-independent siderophore (NIS) synthetase pathway (Figure S1b and Figure S1c) were detected in the genome. The compositions of these putative gene clusters were generally syntenic with those reported in the respective siderophore BGCs in the genomes of other *Armillaria* spp. However, some putative mutations were observed in the BGCs in the genome of strain CMW4456. For instance, the BGC belonging to the NRPS-dependent siderophore pathway in the genome of strain CMW4456 lacked the (Choline/ethanolaminephospho) transferase and the Cellobiose dehydrogenase genes. Additionally, like the gene cluster belonging to *A. cepistipes*, the main biosynthesis gene of the first BGC belonging to the NIS synthetase pathway in the genome of CMW4456 appeared to be a merged gene consisting of genes which encode the IucA/IucC family containing protein and the C2 domain-containing protein. Similarly, the main biosynthesis gene of the second BGC belonging to the NIS synthetase pathway in the genome of CMW4456 appeared to be a merged gene consisting of genes that encode the IucA/IucC family domain containing protein and WH1-domain-containing protein. There were also some putative gene loss and inversion events in the identified siderophore BGCs in the genome of CMW4456. Nonetheless, InterProScan analyses of the amino acid sequences of the main biosynthetic genes in the identified BGCs using InterPro 101.0 ^52^ revealed the presence of the domain architectures characteristic of these proteins.

Our results showed putative expression of the proteins encoded by some of the genes contained in each of the BGCs. These included multiple copies of proteins such as ABC transporter, S-adenosyl-L-methionine-dependent methyltransferase, p-loop domain-containing nucleoside triphosphate, cytochrome P450, aconitate hydratase, and Major Facilitator Superfamily (MFS) transporters in the proteome (Material S1). All of these proteins were commonly expressed in the proteomes under the two experimental conditions. However, the proteins encoded by the main biosynthesis genes were not detected in the proteome or the secretome.

## 4 Discussion

Results obtained in this study revealed enhanced mycelia growth and no significant difference in siderophore biosynthesis, as well as proteomic and secretomic alterations of *Armillaria* sp. strain CMW4456 with iron supplementation. The differentially expressed proteins identified in the proteomes and secretomes were involved in central and secondary metabolism as well as amino acid biosynthesis and growth of *Armillaria* sp. strain CMW4456. We also recorded three putative siderophore biosynthetic gene clusters (BGCs) in the genome and the expression of proteins encoded by some BGC genes in the proteome. These results are discussed in this section.

### 4.1 Iron-Dependent Proteome and Secretome Profiles of *Armillaria* sp. Strain CMW4456

Many proteins (2,360–2,509) were detected in the proteomes obtained under the two conditions (i.e., with the addition of iron and without). This high number of proteins is comparable to that detected for other white-rot basidiomycetes. For example, 2,062 proteins were detected in *Pleurotus ostreatus* proteome^53^ using similar techniques as in our study,^53^ whereas 2,543 proteins were detected in the mycelia of *Hericium erinaceus* using iTRAQ labeling and nano-LC-MS/MS analysis.^54^ On the contrary, fewer proteins (629 to 1,787 proteins) have been detected in the proteomes of *Armillaria mellea* using different approaches.^5,41^ In addition to the presently studied isolate being a different species from that of other studies, different experimental techniques for proteomics have been shown to influence protein detection and other parameters in proteomics research^55^ and may account for this disparity.

Clear separation in the proteome and secretome under both growth conditions in the PCA plot reflected significant protein expression changes between the mycelia and supernatant of *Armillaria* sp. strain CMW4456 as well as iron-dependent alteration of protein production by the mycelia of strain CMW4456 under the study conditions. Proteomic responses to iron have similarly been reported in the fungi, *Aspergillus fumigatus* and *Paracoccidioides brasiliensis*.^12,56^

The range of molecular masses of most of the detected proteins (30–40 kDa) was slightly lower than that reported for the proteome of *Aspergillus flavus* in response to water activity (40–50 kDa).^16^ This discrepancy can be explained by factors including the fact that the two organisms are from different fungal Divisions, only the proteome was considered in the report on *As. flavus*, as well as the different analysis techniques that were used in the respective studies. Fungi belonging to different Divisions (Ascomycota and Basidiomycota) have been shown to possess a diverse repository of enzymes using label-free quantification.^57^

### 4.2 Abundance of Enzymes in the Central Metabolic Pathways is Altered in Strain CMW4456 in Response to Iron Supplementation

Iron supplementation altered the abundance of proteins in central metabolic pathways, including the trichloroacetic acid cycle (i.e., TCA cycle or citrate cycle), the pentose phosphate pathway (PPP), or glycolysis in strain CMW4456. Enzymes involved in the TCA cycle, including dihydrolipoyl dehydrogenase, dihydrolipoyllysine-residue succinyltransferase, fumarate hydratase, pyruvate dehydrogenase E1 component subunit alpha, and pyruvate carboxylase, were increased in abundance in the proteome of strain CMW4456 with iron supplementation. These enzymes were decreased in abundance during iron deprivation in the human pathogenic fungus, *Paracoccidioides brasiliensis*.^56^

Given that several other enzymes involved in the TCA cycle were more abundant in this study, the fact that mitochondrial aconitate hydratase showed no significant change in abundance with iron supplementation in this study suggests that the interconversion of citrate and isocitrate was not altered by strain CMW4456 in response to iron supplementation. Based on transcriptomics analyses, Odoni et al.^58^ showed that an increase in citrate secretion by *Aspergillus niger* under iron-limited conditions is a physiological response and proposed that citrate is an iron siderophore of *A. niger*. Citrate is considered a siderophore in one strain of the bacterial nitrogen-fixing soybean symbiont, *Bradyrhizobium japonicum*.^59^ Our results suggest that the production of citrate for possible use as a siderophore by strain CMW4456 was not altered by the fungus with iron supplementation. This is congruent with our results, which showed no significant difference (*p* = 0.47) in siderophore biosynthesis between PDP– (42.77 ± 0.5 psu) and PDP+ (42.94 ± 1.39 psu). Further research is required to investigate the use of citrate as a siderophore by *Armillaria* spp.

Isocitrate lyase, which is a key enzyme in the glyoxylate cycle (a variation of the TCA cycle), showed no significant change in abundance in both the proteome and secretome of strain CMW4456 with iron supplementation. This enzyme produces glyoxylate and succinate using isocitrate as a substrate.^60^ Isocitrate lyase was decreased in abundance in the proteome of *P. brasiliensis* during iron starvation.^56^ The fact that no significant change in the abundance of isocitrate lyase was observed with or without iron supplementation suggests that iron supplementation of the growth medium with 100 μM FeCl_3_ does not constitute a significant increase in iron availability for strain CMW4456. Increased expression of this enzyme has been reported to be essential for successful colonization and pathogenicity of *Brassica napus* (Canola) by the fungal phytopathogen, *Leptosphaeria maculans*.^61^ Further research would be required to elucidate the role of isocitrate lyase in host colonization as well as pathogenicity and/or virulence of pathogenic *Armillaria* spp.

Proteins such as glucose-6-phosphate 1-dehydrogenase (G6PDH), glucose-6-phosphate isomerase (G6PI), and transketolase, involved in PPP and/or glycolysis, were decreased in abundance in the proteome of strain CMW4566 in the presence of added iron. G6PDH is the first enzyme of the pentose phosphate pathway and is important in resistance to oxidative stress in the fungus *Saccharomyces cerevisiae*.^62^ G6PI was decreased in abundance under copper-induced oxidative stress, while transketolase was commonly expressed by the mycelia of *Penicillium chrysogenum* irrespective of copper concentration in the growth media.^14^ On the contrary, transketolase was increased in abundance in the fungus *Phanerochaete chrysosporium*, in response to the Reactive Oxygen Species (ROS) produced by copper.^11^ Transketolase of the rice blast fungus, *Magnaporthe oryzae*, plays an essential role in facilitating host colonization of rice cells, and invasive hyphal growth was curtailed in transketolase null mutants.^63^ Amide compounds or derivatives have been evaluated for plant and fungal transketolase inhibition.^64^ Therefore, inhibition of transketolase produced by *Armillaria* spp. using novel or existing amide compounds or derivatives may provide avenues for controlling *Armillaria* root- and stem-rot disease.

Taken together, results obtained in this study suggest that the TCA cycle is favored with iron supplementation of the growth medium with 100 μM FeCl_3_. Enhanced metabolism through the TCA cycle contributes to enhanced ATP production.^65^ The recorded protein expression patterns exhibited by strain CMW4456 are congruent with the observed faster culture growth of the fungus in the presence of added iron. Faster culture growth of strain CMW4456 has previously been reported on solid potato dextrose peptone media supplemented with 100 μM FeCl_3_.^31^ Additionally, siderophore biosynthesis of this strain and other strains of *Armillaria* species in PDP with up to 200 μM added FeCl_3_ suggested that *Armillaria* have species- and strain-independent unique requirements for iron.^31^

It is, however, apparent that the addition of 100 μM FeCl_3_ to PDP may be insufficient for significantly increasing the production of enzymes with Fe/S clusters by the fungus, as no significant increase in mitochondrial aconitate hydratase abundance was recorded when cultured in PDP+ compared to PDP–. Other enzymes with Fe/S clusters, such as various NADH dehydrogenases including various subunits of NADH-ubiquinone oxidoreductase, as well as succinate dehydrogenase, also showed no significant change in abundance with iron supplementation in both the proteome and secretome. Such enzymes are mostly involved in redox reactions but also participate in the control of gene expression, oxygen/nitrogen sensing, control of labile iron pool, and DNA damage recognition and repair (reviewed in ref 66). The presence of subunits of mitochondrial membrane-bound proteins such as mitochondrial aconitate hydratase and succinate dehydrogenase [ubiquinone] flavoprotein subunits in the secretome is an interesting finding. Although this may be due to hyphal lysis in the liquid media used for the experiment, this finding would require further research.

Various broad-spectrum fungicides belong to the succinate dehydrogenase inhibitor (SDHI) class. Several phytopathogenic fungi, including basidiomycetes such as *Ustilago maydis*, have been reported to have developed resistance to these fungicides using various mechanisms (reviewed in refs 67–69 ). Although different chemicals have been evaluated against *Armillaria*,^70,71^ to the best of our knowledge, SDHI class fungicides have not been evaluated against *Armillaria*. We propose that existing SDHIs should be evaluated and/or new SDHIs should be developed against *Armillaria* spp. based on the succinate dehydrogenases produced by *Armillaria* spp. These novel fungicides may also provide alternatives to SDHI fungicides, to which fungi have developed resistance.

Overall, reverse expression patterns of almost all of the oxidative stress-related proteins involved in PPP and/or glycolysis were observed in this study compared to the reported expression patterns of these proteins by other fungi under various stress factors including metal stress. Proteomic analyses of oxidative stress-related proteins in *P. brasiliensis* yeast cells were conducted within the period of confirmed cell viability (0–72 h).^56^ Similar evaluations were performed for *S. cerevisiae* after 6 h of incubation^62^ and for *P. chrysogenum* after 1 week of incubation.^14^ In solid media, various strains of *Armillaria* including CMW 4456 were seen to be growing after 3 weeks of incubation.^32^ Therefore, maintaining mycelia viability while obtaining enough biomass and capturing an active stress response, if present, was expected with the 3-week incubation period used in the present study. These results suggest that iron supplementation of PDP at 100 μM for strain CMW4456 under the study conditions does not elicit the stress responses reported for the other fungi to various stress factors. It would be interesting to evaluate the oxidative stress responses of the fungus at different time points.

### 4.3 Amino Acid Biosynthesis, Secondary Metabolism, and Growth are Enhanced with Iron Supplementation

#### 4.3.1 Protein Expression Patterns by Strain CMW4456 in Relation to Amino Acid Biosynthesis and Secondary Metabolism

Proteins associated with amino acid biosynthesis and secondary metabolism showed statistically significant differences in abundance in proteomes obtained with iron supplementation compared to those of the control sample. Among amino acids, leucine biosynthesis as well as the biosynthesis of other branched-chain amino acids such as isoleucine and valine are integral to fungal iron homeostasis and virulence as shown in *As. fumigatus*.^72,73^ Enzymes such as 3-isopropylmalate dehydrogenase increased in abundance in the proteome of strain CMW4456 with iron supplementation. This enzyme is involved in leucine biosynthesis and is required for the biosynthesis of the mycotoxin, pneumocandin in the yeast, *Glarea lozoyensis*.^74,75^ Various compounds have been shown to inhibit 3-isopropylmalate dehydrogenase.^76,77^ The small subunit of acetohydroxy-acid synthase was also increased in abundance in the proteome of strain CMW4456 when cultured in PDP+. This subunit stabilizes, activates, and regulates the activity of acetohydroxy-acid synthase in various organisms using a feedback inhibition mechanism (reviewed in ref 78). Acetohydroxy-acid synthase is essential for the biosynthesis of the branched-chain amino acids, valine, leucine, and isoleucine.^79^ Deletion of the gene which encodes acetohydroxy-acid synthase (*ilv2*) attenuates the virulence of *Candida albicans* and elicits starvation-cidal phenotypes in both *C. albicans* and *Saccharomyces cerevisiae* *ilv2*Δ *m*utants.^80^ Antifungals of *C. albicans* have been developed based on the inhibition of this protein.^81^

Enzymes involved in the biosynthesis of other amino acids were also significantly differentially expressed in the proteome of strain CMW4456 with iron supplementation. Glutamate-5-semialdehyde dehydrogenase and another protein related to probable mitochondrial 4-hydroxy-2-oxoglutarate aldolase are enzymes involved in arginine and proline metabolism. Both enzymes were increased in abundance in the proteome in PDP+ compared to that in PDP–. The former specifically functions in the proline pathway. Like acetohydroxy acid synthase, glutamate-5-semialdehyde dehydrogenase has been used as an antifungal drug target^82^ and should be explored for developing or optimizing antifungal agents against pathogenic *Armillaria* spp. Additionally, homocitrate synthase is an Fe–S protein essential for lysine biosynthesis via the α-aminoadipate (AA) pathway, plays a critical role in mitochondrial function, and is involved in iron metabolism in fungi.^83^ This enzyme was significantly increased in abundance in the proteome of strain CMW4456 with iron supplementation. Both the transcript and protein levels of a homocitrate synthase, LYS4, were increased with iron supplementation in the human fungal pathogen, *Cryptococcus neoformans*, probably to regulate the fitness of the fungus in nutrient-restricted conditions.^83^ Homocitrate synthase has been suggested as an antifungal drug target.^83^ Existing and/or novel antifungals or fungicides that inhibit all the stated enzymes involved in amino acid biosynthesis should be investigated for controlling pathogenic *Armillaria* spp.

All of the amino acid biosynthesis enzymes discussed in this section are also involved in the biosynthesis of secondary metabolites as shown in the KEGG pathways, map01110 and ec01110. Other enzymes involved in the secondary metabolism of strain CMW4456 were discovered to be significantly differentially expressed in the present study, although these proteins do not have any direct linkage to the KEGG database. This shows that more research needs to be conducted on secondary metabolism by *Armillaria* spp. Nevertheless, genetic, genomic, metabolomic, and proteomic-based studies have been conducted to decipher secondary metabolism in *Armillaria* spp. and have revealed a diverse secondary metabolism potential of this genus.^31,32,41,84^ A metabologenomics approach (i.e., the large-scale correlation of gene clusters with metabolites) has been successfully employed to elucidate the secondary metabolism of different actinomycete strains.^85^ This approach should be explored with *Armillaria* spp. to better understand the secondary metabolism of these fungi.

Our findings suggest that iron limitation and antifungal substances that target the biosynthesis pathways of various amino acids can be explored for inhibiting strain CMW4456. This is because enzymes such as 3-isopropylmalate dehydrogenase, acetohydroxy-acid synthase, glutamate-5-semialdehyde dehydrogenase, and homocitrate synthase were increased in abundance in the proteome of strain CMW4456 with iron supplementation in the present study. Research should be undertaken to evaluate the effect of these enzymes on the pathogenicity and/or virulence of *Armillaria* spp. Attenuation of the pathogenicity and/or virulence of strain CMW4456 and potentially other pathogenic *Armillaria* spp. by antifungal substances which target these amino acid biosynthesis pathways should be evaluated and/or optimized. Conversely, this knowledge can be valorized for the biosynthesis of these amino acids and, by extension, secondary metabolite biosynthesis by *Armillaria* spp. for pharmaceutical and other biotechnological applications.

#### 4.3.2 Iron-Dependent Mycelia Growth Enhancement of Strain CMW4456

Mycelia growth of strain CMW4456 was enhanced with iron supplementation at 100 μM. Culture fresh weights of 0.311–0.376 and 0.501–0.556 g were recorded for cultures grown in PDP– and PDP+, respectively. The proteomes obtained under the experimental conditions reflected this increased growth of the fungus with iron supplementation. This is demonstrated by the fact that cellular processes, cells, and cell parts comprised most of the up-expressed DEPs of the proteome of the fungus with added iron as shown by the results of the GO annotations. For example, the growth-related protein, pyruvate dehydrogenase E1 component subunit alpha, increased in abundance with iron supplementation. This enzyme functions in pyruvate metabolism and has been reported to contribute to the long-term survival of fission yeast.^86^ Moreover, the microtubule binding protein was increased in abundance in the proteome by strain CMW4456 with iron supplementation. This enzyme is involved in microtubule cytoskeleton organization.^87^ The microtubule cytoskeleton is essential for filament integrity, polarized growth, transport of organelles and vesicles, and spindle assembly. The microtubule cytoskeleton is therefore required for normal cell morphogenesis in various organisms, including the human fungal pathogen, *Cr. neoformans*,^87^ and Basidiomycota, such as the phytopathogen *U. maydis* (reviewed in ref 88). A member of the microtubule’s building block, tubulin alpha chain, also increased in abundance in the proteome of strain CMW4456 with iron supplementation. These proteins play a role in nuclear migration and positioning in filamentous fungi (reviewed in ref 89). Both DNA replication licensing factor MCM6 and a protein related to protein kinase DBF2 were also increased in abundance in the proteome with iron supplementation. These proteins are involved in DNA replication as a DNA unwinding enzyme (DNA helicase)^90^ and required during anaphase and/or telophase steps in the cell cycle,^91^ respectively. These enzymes may be involved in the culture growth of strain CMW4456. The exact effects of these enzymes on the culture growth of strain CMW4456 and other strains of *Armillaria* spp. should be investigated to better understand the biology of these organisms and to eventually develop more effective control strategies for the phytopathogenic strains or enhance the growth of these fungi for other applications.

### 4.4 Iron Supplementation Does Not Alter Oxidative Stress Response of Strain CMW4456

Supplementation of PDP medium with 100 μM FeCl_3_ did not alter the oxidative stress response by strain CMW4456. This is contrary to the proteomic and secretomic responses observed for various organisms in response to metal concentrations.^10,11,14,18,92–94^ Oxidative stress causes Reactive Oxygen Species (ROS) production and can result in cellular damage (i.e., protein degradation, DNA damage, membrane peroxidation, and apoptosis) when ROS are in high concentrations.^95^ Therefore, organisms utilize various mechanisms for responding to oxidative stress to ensure correct redox balance. These mechanisms include the assembly of antioxidant enzymes, metal-chelating proteins or molecules, and free radical scavengers.^14,28^

Organisms increase the production of various enzymes under oxidative stress caused by abiotic factors, including exposure to high metal concentrations. These enzymes include aldehyde dehydrogenase, ATP phosphoribosyltransferase, ATP synthase subunits, glutamate–cysteine ligase, glutathione reductase, glutathione synthetase, glutathione S-transferase, heat shock proteins, NADPH cytochrome P450 reductase, NADH-ubiquinone oxidoreductase, saccharopine dehydrogenase, superoxide dismutase, thioredoxin reductase, and trehalase.^10,11,14,15,18,19,41,92–94^ However, glutathione S-transferase was identified under both iron-replete and iron-deplete conditions in *As. fumigatus* microsomal extracts.^12^

Most of the implicated oxidative stress response-related enzymes play a significant role in the glutathione (GSH) biosynthesis pathway (reviewed in refs 96,97 ). Glutathione is an important antioxidant which can sequester metals to provide tolerance to the cellular components of organisms against heavy metals.^98^ Overexpression of proteins such as ATP phosphoribosyltransferase and saccharopine dehydrogenase, involved in histidine and glutamate biosynthesis, respectively, indicates an increase in amino acid metabolism potentially for the replacement of misfolded proteins caused by ROS.^10,14,94^ Saccharopine dehydrogenase was reported to increase in abundance in the proteome of an *Armillaria mellea* isolate in response to H_2_O_2_-induced stress.^41^ Superoxide dismutase (SOD) functions in the detoxification and metabolism of ROS to prevent oxidative damage and plays a role in fungal-host (including plant-pathogen) interactions.^30,99^ SOD has been reported to increase in response to cadmium exposure in the mycelia of the basidiomycete, *Paxillus involutus*, grown in liquid medium.^15^ This enzyme has also been shown to increase significantly in *Candida* spp. during hyphal morphogenesis, and in response to a burst in ROS and iron starvation stress.^30^ Cu-only SODs have been recommended as a target for antifungal agents.^30^ Additionally, the accumulation of trehalase is implicated in the accumulation of the disaccharide, trehalose, which functions to prevent the aggregation of denatured proteins and scavenging free radicals.^100^ Another enzyme, Am16706, which showed homology to insulin-degrading enzyme (IDE), was reported to be decreased in abundance in *A. mellea* in response to H_2_O_2_-induced stress due to purported inactivation and subsequent degradation of Am16706 by H_2_O_2_.^41^

The proteins usually reported to increase in abundance in response to oxidative stress and/or metal toxicity, including the enzymes involved in the GSH biosynthesis pathway as well as ATP phosphoribosyltransferase, saccharopine dehydrogenase, SOD, trehalase, and IDE, generally showed no significant difference in abundance in the proteome of strain CMW4456 under the two experimental conditions in this study. Hence, the addition of iron at 100 μM to the growth medium did not alter the oxidative stress response of strain CMW4456. This evidently further supports our hypothesis that iron supplementation of PDP at 100 μM is not a toxic concentration of iron for this strain of *Armillaria* under the study conditions.

### 4.5 Siderophore BGCs Identified, and Expression of Some Proteins Encoded by Genes in the BGCs

The siderophore BGCs detected in this study largely showed microsynteny, in terms of gene composition and orientation, as well as conserved domain architectures of the main biosynthetic genes compared to the previously reported putative siderophore BGCs in other strains of *Armillaria* spp.^31,32^ This concurs with the proposition that these BGCs are largely conserved in the genomes of *Armillaria* spp., potentially differing in their siderophore biosynthesis, may produce siderophores which are different from the siderophores produced by other fungi, and may be involved in the evolutionary success of members of the genus, as previously reported.^31,32^ The few modifications observed in the BGCs within the genome of strain CMW4456 compared to the BGCs of the genomes of the other *Armillaria* spp. potentially demonstrate that the BGCs in the genome of strain CMW4456 may differ in their siderophore biosynthesis compared to the other *Armillaria* spp. Strain CMW4456 has been shown to differ in *in vitro* biosynthesis of siderophores using the universal and the split CAS assays compared to other strains of *Armillaria* spp., as the strain showed no change of the blue CAS media to orange.^32^ The quality metrics of the draft genome of strain CMW4456 are comparable to the genome quality metrics of genomes of isolates of other *Armillaria* species.^33^ The genome was also appropriate for the proteogenomic analyses conducted in the present study, as all the siderophore BGCs were identified and the gene contents of the respective siderophore BGCs were generally seen to be complete. Nevertheless, the modifications observed in the BGCs within the genome of strain CMW4456 compared to the BGCs of the other genomes can be further studied using techniques such as targeted mutagenesis coupled with targeted metabolomics and functional assays as well as improved reference genomes and/or telomere-to-telomere genome assemblies. These suggested studies will enhance genome annotation and enable more robust identification of the genes or mutations in the BGCs in the respective genomes. Such studies are relevant because siderophore biosynthesis plays a crucial role in fungal iron homeostasis, environmental adaptation, and pathogenicity.^23,24,101,102^

As there were multiple copies of some of the proteins encoded by the genes in these BGCs and the expression of the main biosynthesis genes was not recorded in this study, it was impossible to draw concrete conclusions about the expression of the genes in these BGCs. This may be due to some of the limitations of proteomics as outlined by Schubert et al.^103^ Hence, it would be relevant to use techniques such as reverse transcription-quantitative polymerase chain reaction (i.e., RT-qPCR) together with targeted metabolomics and functional assays to investigate the expression and functions of the genes in these BGCs as previously described.^104^ Nonetheless, the fact that the proteins encoded by some of the genes in these putative siderophore BGCs were commonly expressed in the proteomes under the two study conditions concurs with our results, which showed no significant difference in siderophore biosynthesis by strain CMW4456 under the experimental conditions.

## 5 Conclusions

In this study, the proteome and secretome of an *Armillaria* strain from Africa were investigated for the first time. The comparative proteomic and secretomic study conducted demonstrated that a change in protein expression by strain CMW4456 was induced with the supplementation of the growth medium with 100 μM FeCl_3_. These proteomic and secretomic changes recorded were involved in the central and secondary metabolism and growth. The supplementation of PDP with 100 μM FeCl_3_ was apparently not sufficient to support all molecular and biological processes of the fungus. The difference in protein expression is not the same as one would expect if the supplement caused oxidative stress to the fungus. The proteogenomics approach used in this study confirmed the putative presence of all the previously reported putative siderophore BGCs in the genomes of other *Armillaria* spp. in the genome of strain CMW4456. These include one BGC belonging to the NRPS-dependent siderophore pathway and two BGCs belonging to the NRPS-independent siderophore synthetase pathway. However, this approach could not conclusively prove the expression of the genes in these BGCs. Hence, other techniques, such as RT-qPCR with targeted metabolomics and functional assays, as well as efforts to obtain more contiguous and/or telomere-to-telomere genome assemblies, would be relevant to evaluate the expression and functions of the genes in the BGCs. Nonetheless, the results of this study give a deeper understanding of the biology of this strain of *Armillaria* in terms of iron homeostasis using the gel-free and label-free shotgun LC-MS/MS approach. Results from this study also identify potential targets for inhibition and attenuation of the pathogenicity and/or virulence of strain CMW4456. These include the investigation and application of efficacious and sustainable technologies such as existing and/or novel SDHIs and transketolase inhibitors, as well as antifungal substances which will target iron bioavailability to the fungus and the biosynthesis of proline, lysine, and the branched-chain amino acids valine, leucine, and isoleucine by the fungus. The knowledge generated in this study can also be applied to improve bioprocesses for enhanced mycelia growth of *Armillaria* spp. as well as the biosynthesis of various amino acids and secondary metabolites by *Armillaria* spp. for pharmaceutical and other biotechnological applications.

## Supplementary Material





## Data Availability

Data reported in this manuscript and all other supporting information available in the manuscript are openly available online. The mass spectrometry proteomics data have been deposited to the ProteomeXchange Consortium (http://proteomecentral.proteomexchange.org) via the PRIDE^104^ partner repository with the data set identifier PXD068883. Publicly available genomes and RNA sequences of *Armillaria* species were analyzed for the proteogenomic study. These data can be found at https://mycocosm.jgi.doe.gov/mycocosm/species-tree/tree;05h0Ue?organism=physalacriaceae and at NCBI GenBank with accession number JANDKJ000000000.

## Notes

Figure S1: Synteny maps of putative siderophore BGCs; a pdf document containing synteny maps of the three putative siderophore BGCs in the genome of strain CMW4456 compared to those of putative siderophore BGCs in the genomes of other *Armillaria* spp. (PDF)
Material S1: Quantitative differences in the protein expression profiles in the respective sample groups; an excel sheet which contains quantitative differences in the protein expression profiles in the respective sample groups; the definitions of the headings can be found in the sheet named “Definitions; all proteins discussed in the manuscript have been highlighted (XLSX)

## ‡

Department of Biology, Stanford University, Stanford, CA, 94305 United States
