# Supplementary material for: Proteomic and Secretomic Response of an African Armillaria Species to Iron
Source: J Proteome Res. 2026 Feb 20;25(3):1611–25. doi: 10.1021/acs.jproteome.5c00979 (PMC12973365; doi:10.1021/acs.jproteome.5c00979)
Supplement: Supplementary file 1 [file pr5c00979_si_001.pdf]

Proteomic and secretomic response of an African *Armillaria* species to iron

Deborah L. Narh[1,2], Brenda D. Wingfield[1], Martin P. A. Coetzee[1*]

[1]Department of Biochemistry, Genetics and Microbiology, Forestry and Agricultural Biotechnology Institute (FABI), Faculty of Natural and Agricultural Sciences, University of Pretoria, Pretoria 0002, South Africa

Present address:

[2]Department of Biology, Stanford University, Stanford, CA, 94305 United States

Email addresses: DLN: dlnarh@stanford.edu ; BDW: Brenda.wingfield@ fabi.up.ac.za; MPAC[*]: martin.coetzee@fabi.up.ac.za

Table of Contents

Figure S1 - Synteny maps of the three putative siderophore biosynthetic gene clusters (BGCs) in the genome of strain CMW4456 compared to those of putative siderophore BGCs in the genomes of other *Armillaria* spp. (pdf)

Material S1 - Quantitative differences in the protein expression profiles in the respective sample groups. (xlsx)

(a) Synteny map of putative NDSS BGC

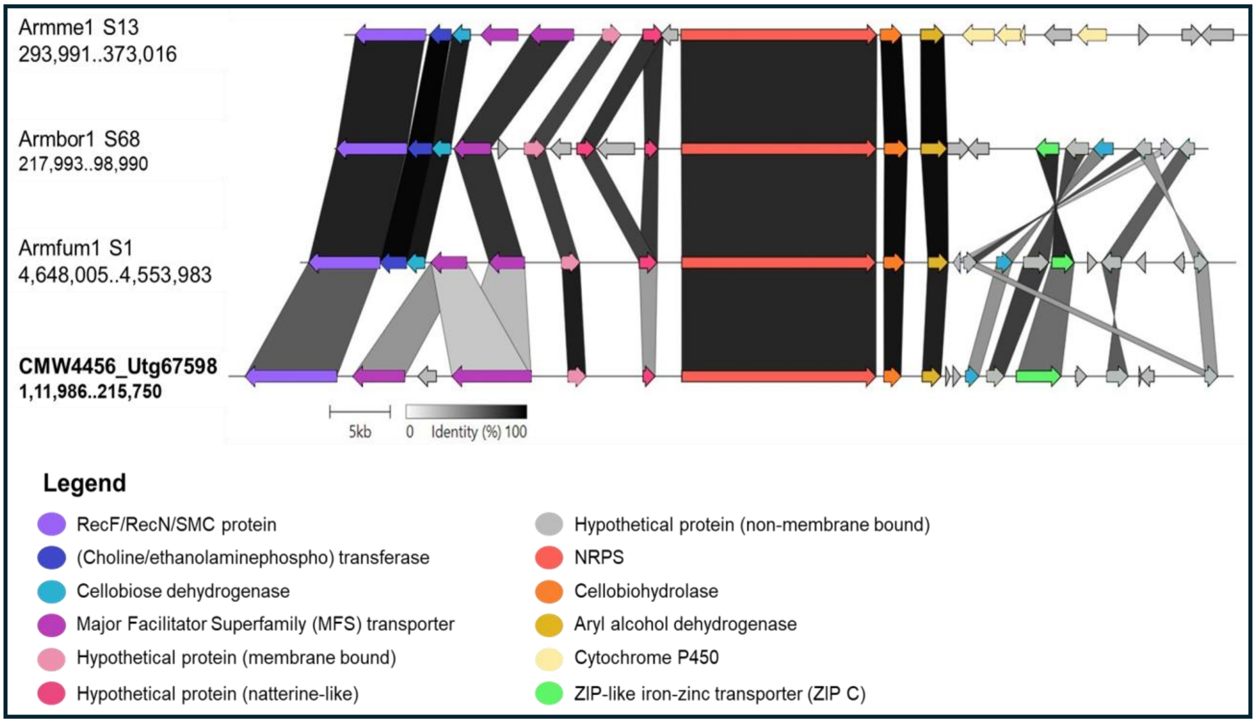

(b) Synteny map of first NIS synthetase BGC

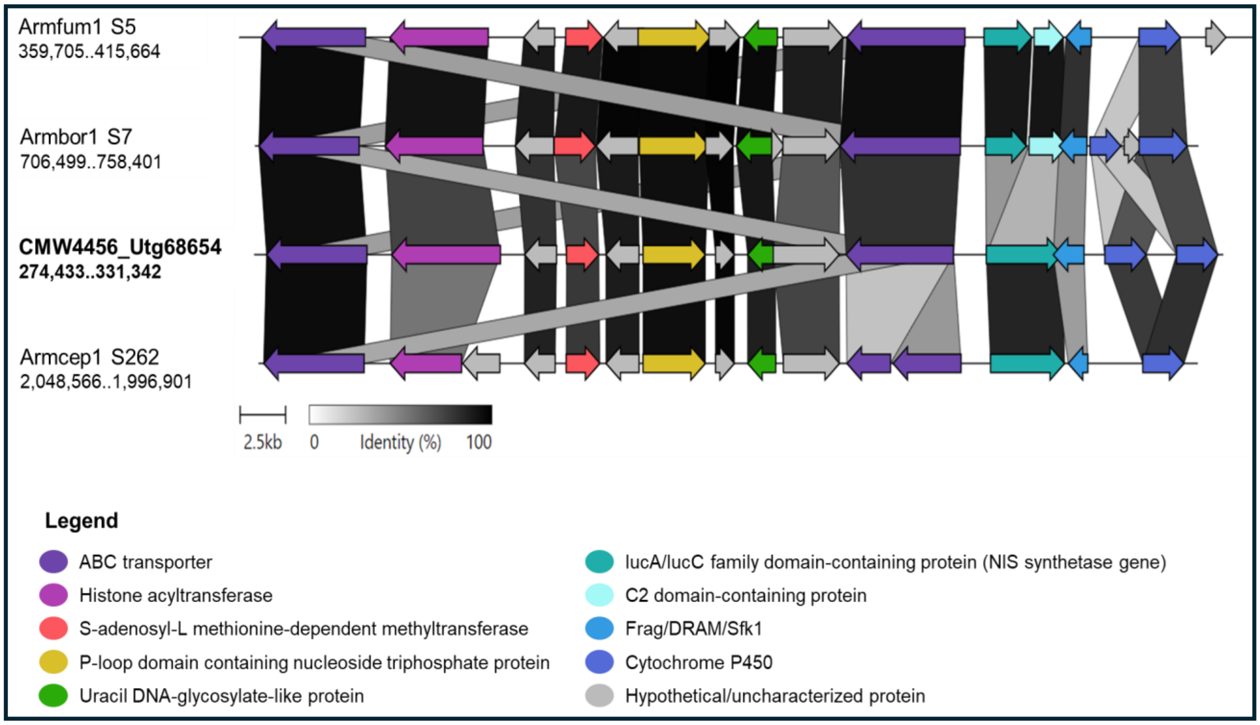

(c) Synteny map of second NIS synthetase BGC

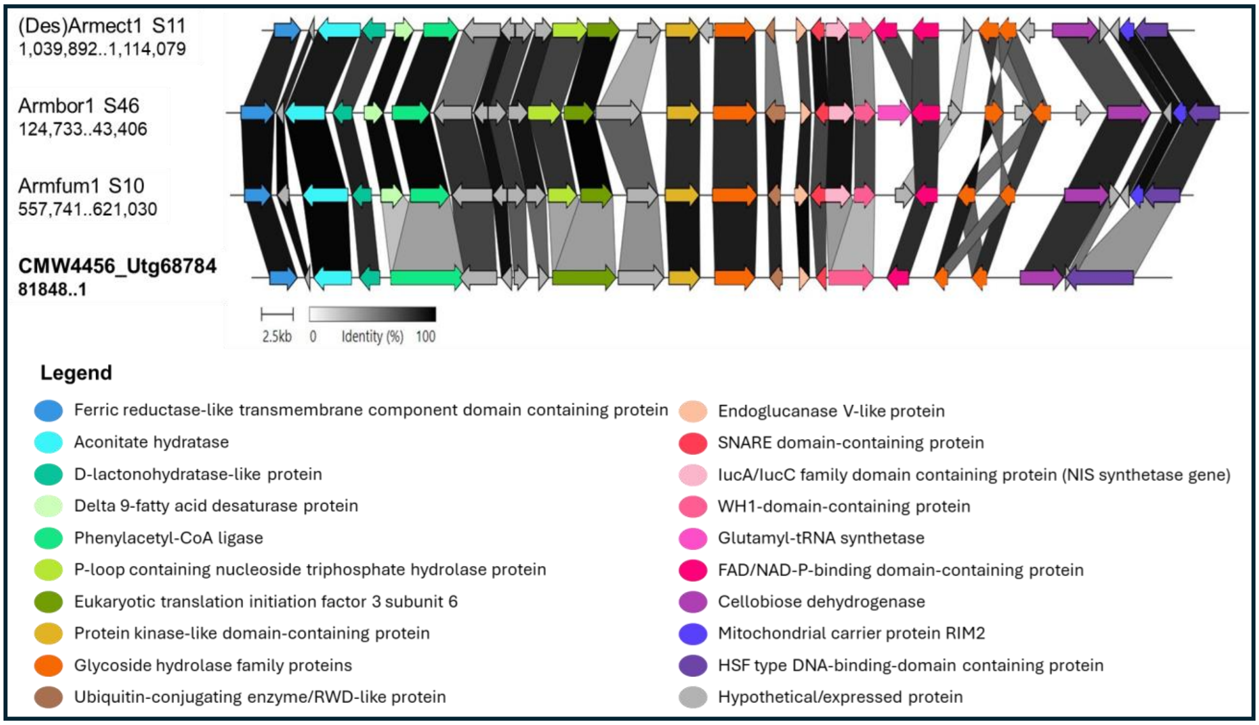

Figure S1: **Siderophore BGCs synteny maps in annotated genome of *Armillaria* sp. strain CMW4456 compared with the reported putative biosynthetic gene clusters (BGCs) in the genomes of other *Armillaria* spp.** (a) Synteny map of putative NDSS BGC: Synteny maps of non-ribosomal peptide synthetase (NRPS)-dependent siderophore synthetase gene clusters in annotated genome of *Armillaria* sp. strain CMW4456 compared with the reported putative BGCs in the genomes of other *Armillaria* spp. (b) Synteny map of first NIS synthetase BGC: Synteny maps of first NRPS-independent siderophore (NIS) synthetase gene clusters in annotated genome of *Armillaria* sp. strain CMW4456 compared with the reported putative BGCs in the genomes of other *Armillaria* spp. (c) Synteny map of second NIS synthetase BGC: Synteny maps of second NRPS-independent siderophore (NIS) synthetase gene clusters in annotated genome of *Armillaria* sp. strain CMW4456 compared with the reported putative BGCs in the genomes of other *Armillaria* spp. In each figure, each row represents a BGC in the respective genome (represented as the species code of the respective species). Numbers following the species code are the scaffolds on which the BGCs are located. Numbers below the species codes are the sequence locations of the BGCs within the scaffolds. Different colors and orientation of arrows represent different putative genes as determined by tBLASTn searches and direction of transcription respectively. Orthologous genes are identically colored. Shadings between rows indicate the similarity between the genes in the adjoining BGCs. NDSS = non-ribosomal peptide synthetase (NRPS)-dependent siderophore synthetase; NIS = NRPS-independent siderophore; Armbor1 = *A. borealis* strain FPL87.14 v1.0; Armcep1 = *A. cepistipes* strain B5; Armfum1 = *A.*

fumosa strain CBS 122221 v1.0; Armme1 = *A. mellea* strain ELDO17 v1.0; (Des)Armect1 = *Desarmillaria ectypa* strain FPL83.16 v1.0
